# Directing cell therapy to anatomic target sites *in vivo* with magnetic resonance targeting

**DOI:** 10.1038/ncomms9009

**Published:** 2015-08-18

**Authors:** Munitta Muthana, Aneurin J. Kennerley, Russell Hughes, Ester Fagnano, Jay Richardson, Melanie Paul, Craig Murdoch, Fiona Wright, Christopher Payne, Mark F. Lythgoe, Neil Farrow, Jon Dobson, Joe Conner, Jim M. Wild, Claire Lewis

**Affiliations:** 1 Departments of Infection and Immunity, University of Sheffield, Sheffield S102RX, UK; 2 Department of Psychology, University of Sheffield, Sheffield S102RX, UK; 3 Department of Oncology, University of Sheffield, Sheffield S102RX, UK; 4 Unit of Oral & Maxillofacial Medicine & Surgery, University of Sheffield, Sheffield S102RX, UK; 5 Centre for Advanced Biomedical Imaging, Division of Medicine, University College London, London WC1E 6DD, UK; 6 Institute for Science and Technology in Medicine, Keele University, Stoke-on-Trent ST4 7QB, UK; 7 Department of Biomedical Engineering, University of Florida, Gainesville, Florida 32611, USA; 8 Virttu Biologics, Glasgow, Glasgow City G51 4TF, UK; 9 Cardiovascular Sciences, University of Sheffield, Sheffield S102RX, UK

## Abstract

Cell-based therapy exploits modified human cells to treat diseases but its targeted application in specific tissues, particularly those lying deep in the body where direct injection is not possible, has been problematic. Here we use a magnetic resonance imaging (MRI) system to direct macrophages carrying an oncolytic virus, Seprehvir, into primary and metastatic tumour sites in mice. To achieve this, we magnetically label macrophages with super-paramagnetic iron oxide nanoparticles and apply pulsed magnetic field gradients in the direction of the tumour sites. Magnetic resonance targeting guides macrophages from the bloodstream into tumours, resulting in increased tumour macrophage infiltration and reduction in tumour burden and metastasis. Our study indicates that clinical MRI scanners can not only track the location of magnetically labelled cells but also have the potential to steer them into one or more target tissues.

## Abstract (web summary)

Cell therapy requires the targeting of cells to specific sites in the body. Here Muthana *et al.* use a standard MRI scanner to direct oncolytic macrophages, labelled with magnetic nanoparticles, to primary and metastatic tumour sites in mice, and demonstrate that this leads to reduced tumour growth.

## Graphical abstract

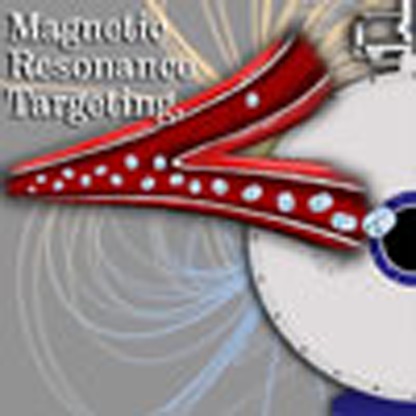

Advances in our understanding of the molecular mechanisms underpinning major diseases have led to the development of a wide array of cell-based therapies to deliver a therapeutic agent such as a protein or virus, or a modified, repopulating stem cell^1^. When the disease is not confined to one site in the body, or is in a tissue inaccessible by direct injection of cells, such cell-based therapies have to be administered systemically.

Previous studies have shown that magnetic particles or cells loaded with super-paramagnetic iron oxide nanoparticles (SPIOs) can be injected systemically and attracted to a target tissue in mice by the application of a local external magnet^2^,^3^,^4^,^5^,^6^. Indeed, we have previously showed that SPIO-loaded human macrophages could be attracted from the circulation into tumours in mice using such an approach^7^. However, this approach can only be applied to superficial target tissues. While localized magnetic field gradients could be achieved in deeper tissues using implanted ferromagnetic stents^8^, this necessitates invasive surgery.

An exciting, alternative approach is magnetic resonance targeting (MRT) that uses the magnetic field gradient coils inherent to all magnetic resonance imaging (MRI) systems, to steer ferromagnetic particles (or cells containing them) to a target site^4^. We have previously shown that MRI could be used to steer iron-labelled human peripheral blood mononuclear cells in a vascular model^7^, and early studies in pigs demonstrated this concept by steering a 1.5-mm ball bearing a distance of 5 cm inside the right carotid artery of the animal using the gradient coil currents of a standard 1.5 T MRI system^9^,^10^.

Bone marrow-derived cells are increasingly being used in cell-based therapies for such diseases as infarcted myocardium^11^, spinal cord injury^12^, cerebral ischaemia^13^ and degenerative diseases such as Parkinson’s disease^14^, Alzheimer’s disease^15^ and cancer^16^,^17^,^18^. In the latter disease, numerous clinical trials have administered bone marrow-derived cells systemically in an attempt to treat malignant tumours, including T cells^19^,^20^, dendritic cells^21^, macrophages^22^,^23^ and stem cells^24^. However, only a small proportion of these cells subsequently locate to the tumour site, with many found subsequently in other tissues. This lack of targeting not only reduces the therapeutic efficacy but also increases the risk of side effects.

When macrophages were found to accumulate in large numbers in avascular hypoxic/necrotic areas of such tissues in mice and humans^17^,^25^,^26^, we suggested that these cells could be used to deliver therapeutic agents such as oncolytic viruses (OVs) to these poorly vascularized, and therefore relatively inaccessible, areas of tumours^17^. In the present report, we show that MRT can be used to increase the number of OV-loaded macrophages in primary and metastatic tumours in mice. Importantly, MRT markedly increased the anti-tumour effects of this macrophage virotherapy. Our results suggest that it is possible to use a standard MRI scanner to non-invasively steer cells to both primary and secondary tumours, and, so, in theory, this approach could be used to steer any cell-based therapy to its target site(s) within the body.

## Results

### MRT of magnetic cells into three-dimensional tumour spheroids

**Figure 1:**
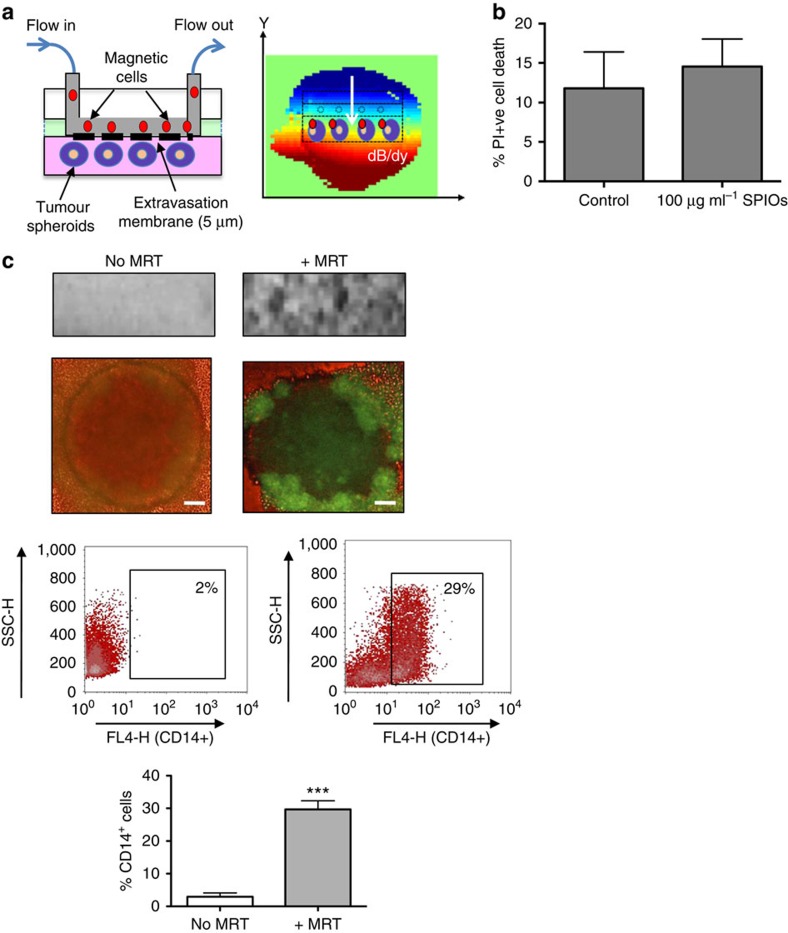
MRT using a novel transendothelial migration (TEM) flow assay. We have designed a flow chamber that can accommodate three-dimensional (3D) tumour spheroids as well as a vascular endothelial layer. The flowing ‘magnetic cells’ will therefore need to cross the vascular barrier before entering a 3D tumour simulating the passage of cells across endothelial cells in a blood vessel wall (**a**, left panel). The TEM flow chamber is placed in the isocentre of an MRI scanner with a spherical (6-mm diameter) homogenous 7 T magnetic field. We applied a pulsed gradient (50% of max) with strength of 300 mT m^−1^ in the (−*y*) gradient direction. The resulting heterogeneous magnetic field (d*B*/d*y* field) can steer magnetic particles towards the tumour spheroids for increased uptake (**a**, right panel). Cell viability following SPIO uptake was not compromised as assessed by flow cytometry for propidium iodide uptake (**b**). Uptake was confirmed by a distortion in the MRI image and a loss of signal compared with when no MRT was applied (**c**, upper panel). Corresponding fluorescent images of whole spheroids infiltrated with macrophages carrying a reporter adenovirus (Ad-CMV-GFP) are shown in (**c**, second panel). Scale bar, 100 μm. Flow cytometry of enzymatically dispersed spheroids revealed that the number of magnetic cells infiltrating spheroids (% of all cells present in spheroids that were CD14+) was significantly increased when a gradient was applied (**c**, lower panels). All images and data (means±s.e.m.) are derived from six independent experiments. Comparisons between the groups were performed using two-tailed unpaired Student’s *t*-test ***P=0.0001, compared with no MRT group.

Before applying MRT techniques *in vivo*, we first established that a pre-clinical 7 T MRI system fitted with a 600 mT m^−1^ gradient coil (limited to ∼300 mT m^−1^ for this study) set could generate substantial actuation forces on magnetic macrophages *in vitro* by steering them across an endothelial layer into three-dimensional human multi-cellular tumour spheroids (MTS). To do this, we designed a transendothelial migration (TEM) flow chamber in which human macrophages circulated across the surface of a perforated membrane coated with a layer of human vascular endothelial cells, thereby mimicking flow in tumour venules. MTS were cultured in a non-adherent chamber below the membrane (Fig. 1a). Human macrophages infected with a green fluorescent protein (GFP) reporter adenovirus (Ad-CMV-GFP) were loaded with SPIOs (1.18±0.3 μg ml^−1^)^7^ and then steered across the membrane into MTS when the chamber was placed in the isocentre of a pre-clinical MRI system. SPIO uptake did not affect macrophage viability (Fig. 1b).

MRT experiments used a pulsed magnetic field gradient (2-ms on, 7-ms off, 50% strength ∼300 mT m^−1^ (ref. 4)) for 1 h in the direction of the spheroids (Fig. 1a) with an effective additional magnetic field offset, *B*_off_∼+1.5 mT around the MTS site. In control conditions, samples were exposed to the magnetic field of the scanner but gradients were not pulsed. Following MRT, we found a T_2_*-weighted signal loss indicating higher concentration of iron in comparison with the control samples for MRT samples (*n*=6) (Fig. 1c, upper panel). GFP-expressing macrophages were also clearly visible within MTS (Fig. 1c, second panel) and flow analysis further confirmed macrophage uptake with significantly more viable infiltrating CD14^+^/propidium iodide^−^-expressing macrophages with MRT (29.7±2.6%) than without (2.9±1.8%; Fig. 1c, lower panel).

### MRT improves tumour uptake of magnetic cells *in vivo*

**Figure 2:**
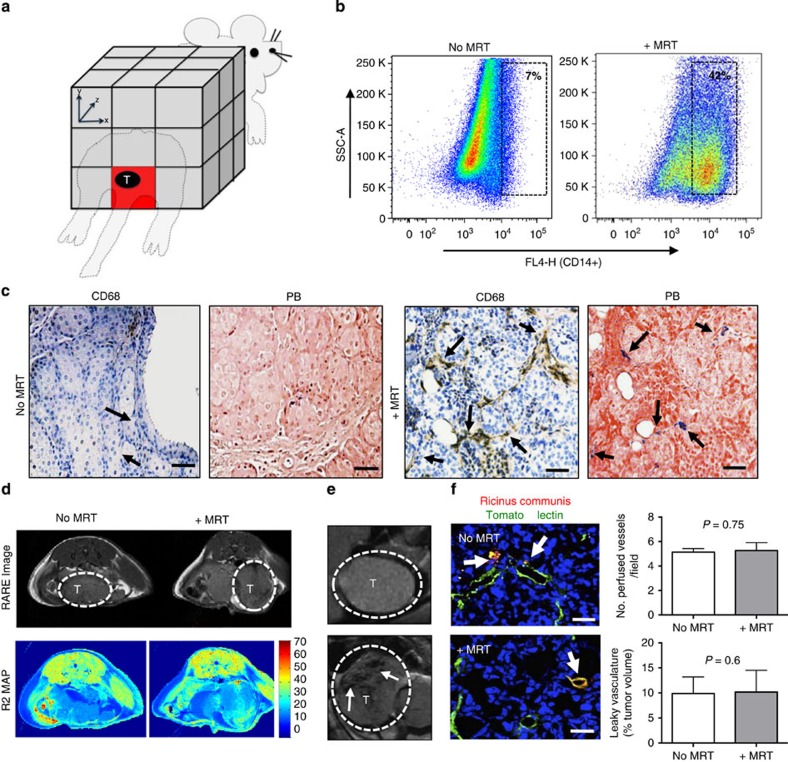
Magnetic macrophages were steered into primary prostate tumours using MRT. (**a**) Schematic of targeted regions using imaging gradients for MRT. We applied a −*y* gradient equally across the animal to target the location of the prostate as depicted (red box). Three million magnetically labelled macrophages were then administered to mice via i.v. injection and anaesthetised mice were then placed into the isocentre of a 7 T MRI scanner. Subjects were split into two groups with *n*=5 mice per group. Group 1 were imaged after 1 h (no MRT). Group 2 underwent MRI targeting. Post mortem, we confirmed the increased levels of human macrophage uptake by (**b**) FACS analysis of collagenase-treated tumours 1 h after MRI targeting, and (**c**) histological staining of paraffin wax-embedded tumour sections with an anti-human CD68 antibody and Prussian blue (CD68-positive macrophages are brown and SPIO-positive macrophages are blue: see arrows). Representative RARE images from five mice per group and R2 maps for each group are shown in **d** and **e**. T, tumour; scale bar, 200 μm. (**f**) The number of perfused vessels in tumours in mice that underwent MRT compared with mice with no MRT was determined by perfusion with tomato lectin (green). Arrows point to areas vascular leakage. Mean±s.e.m., *P*=0.75, Student’s *t*-test; analysis of tumours from *n*=5 mice per group and 5–10 fields of view (FOV) per tumour. The total volume of leaky vasculature does not differ between tumours in the two groups of mice (mean±s.e.m.; *P*=0.6, Student’s *t*-test; analysis of tumours from three mice per group and 5–10 FOV per tumour). Scale bar, 34 μm.

We then investigated whether such an MRI gradient system could be used to steer magnetic macrophages to tumours *in vivo* (Fig. 2). Three million SPIO-loaded macrophages were administered intravenously to mice bearing orthotopic primary and metastatic (lung) prostate tumours. A pulsed magnetic field gradient^4^ was applied for 1 h, in the direction of the prostate (Fig. 2a), with an effective magnetic field offset, *B*_off_∼+7 mT on top of the static magnetic field of the scanner (*B*_0_=7 T). The control group was exposed to the static magnetic field of the scanner in the absence of the steering gradients (no MRT).

MRT significantly (unpaired student *t*-test, *P*=0.0001) increased uptake of SPIO-loaded macrophages in primary prostate tumours (42.2±2.5%) compared with the control group who did not undergo MRT (7.17%±0.8) (Fig. 2b). Moreover, we observed these SPIO+ human macrophages were distributed throughout tumours with very few signs of cell clumping in the tumour vasculature following MRT as seen by labelling sequential sections of tumours using an antibody against human CD68 (a pan macrophage marker) and a histological stain for iron (Prussian blue) (Fig. 2c). This was also confirmed by immunofluorescence staining where tumour cells are labelled with the anti-GFP antibody (green) and tumour infiltrating human macrophages with anti-CD68 (red) (Supplementary Fig. 1a). MRI steering of macrophages did not adversely affect the tumour vasculature (Supplementary Fig. 1b); we examined the morphology and integrity of every CD31+ blood vessel in each of the five tumours in these two groups and found no differences between them. We could not see signs of endothelial cell disruption nor were there any signs of blood clotting (for example, platelet aggregation) in, or on, the abluminal side of blood vessels after MRI targeting. In the multi-echo rapid acquisition with relaxation enhancement (RARE) magnetic resonance images of tumours, little difference can be seen between the MRT and no MRT groups (Fig. 2d). This is most likely due to the blood pool iron content per voxel. However, a marked difference between SPIO-injected and non-injected subjects is evident in the T2-weighted long echo time (TE) images, with the loss in signal intensity within the tumour indicating the presence of high concentrations of iron (Fig. 2e). In an effort to assess the increased uptake of magnetic macrophages *in vivo*, we used magnetic resonance relaxometry to measure the magnetic resonance transverse relaxation decay rate (*R*_2_) in tumours in both the groups. *R*_2_ measurements were 21.8 s^−1^ for the MRT group and 18.8 s^−1^ for the control group. Normal *R*_2_ decay rate of tumour tissue without the presence of any SPIOs is also included for comparison (10.5 s^−1^). The increased *R*_2_ decay rate indicated increased iron uptake for the MRT group, suggesting that it is possible to assess the uptake with MRI, as seen with the post-mortem analysis (Supplementary Fig. 1c). The difference in targeted and non-targeted *R*_2_ values was used to estimate the optimal TE for analysing signal differences with spin echo-based MRI sequences (TE of 60 ms). Using this TE, MRT leads to a 10% decrease in signal over the time-matched controls.

Additional controls included tumour-bearing mice: (i) with unlabelled macrophages and MRT and (ii) with unlabelled macrophages without MRT. For these control groups, we detected very few macrophages within tumours as confirmed by MRI (Supplementary Fig. 1d) and flow cytometry of enzymatically dispersed tumours (Supplementary Fig. 1e). Of note, we detected virtually no human CD68+ macrophages in other tissues including the liver (<2% of all cells per tissue section), spleen (<1%) and kidneys (none detected) (Supplementary Fig. 2).

**Figure 3:**
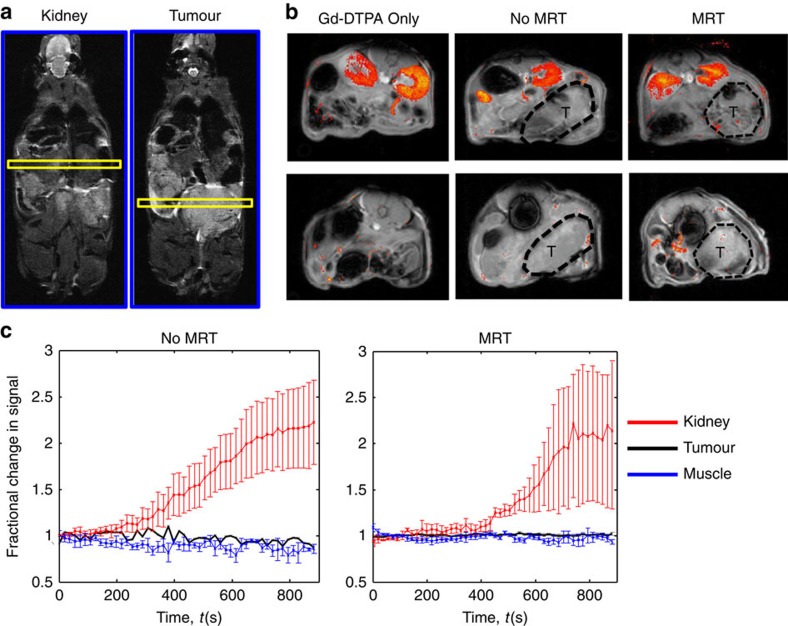
MRT of magnetic cells into tumours does not affect vasculature. Representative maps of Gadolinium pooling are shown for each group of mice where *n*=3 mice per group. The position of the slices (yellow) from which the MRI images were taken is shown—one for the kidneys and the other the tumour (**a**). In all cases, the T1-weighted images show higher signal intensity in the kidneys (**b**; upper panel) than in the prostate tumours (**b**; lower panel), where gadolinium pooled within the urinary system of the mice following administration. (**c**) Regions of interest were taken in the kidney, tumour and muscle tissue and time series analysis of contrast agent pooling is shown for the MRT group and time matched ‘no MRT’ group. Both muscle and tumour tissue show no apparent change in magnetic resonance signal intensity over time compared with the kidney, providing further evidence that the vasculature has remained intact following MRT therapy. All data are means±s.e.m. from *n*=3 mice per group.

To further investigate whether macrophage delivery to tumours by MRT disrupted the function or integrity of the tumour vasculature, vascular perfusion and permeability was determined using intravenous (i.v.) tomato lectin and *Ricinus communis* agglutinin I staining, respectively^27^. We could not detect any differences in the number of perfused vessels or vascular leakage between the no MRT and +MRT groups (Fig. 2f). This was confirmed by estimating vascular permeability to gadolinium-diethylenetriaminepentaacetic acid (Gd-DTPA) by dynamic contrast-enhanced MRI (Fig. 3).

### MRT of magnetic cells to pulmonary metastasis

**Figure 4:**
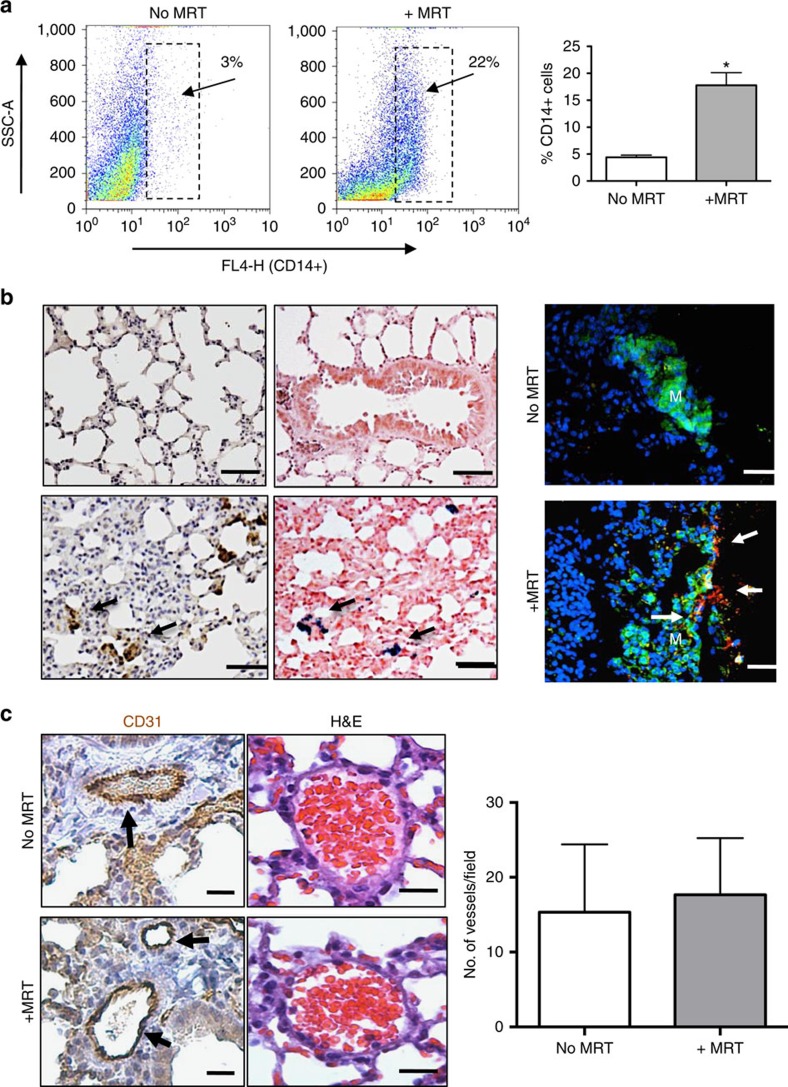
Magnetic macrophages were steered into pulmonary metastasis using MRT. Short-pulsed magnetic gradients were used to steer SPIO-loaded macrophages towards the lungs. (**a**) FACS analysis of collagenase-treated lungs showed significantly more human CD14+ macrophages were present in lungs with than without MRT. Analysis of tumours from *n*=3 mice per group where data are mean±s.e.m., Student’s *t*-test *P<0.01 compared with non-magnetic resonance targeted lungs. (**b**) Representative images from *n*=3 mice per group are shown, this revealed increased immunostaining for human CD68 and Prussian blue in lung sections (CD68+ cells are brown and SPIO+ cells blue: see arrows). Scale bar, 75 μm. This was also confirmed in immunofluorescence-stained sections using an anti-GFP antibody/green and CD68/red (scale bar, 75 μm, M, metastasis and arrows point to CD68-positive cells). (**c**) Immunostaining with CD31 (scale bar, 50 μm) and haematoxylin and eosin (scale bar, 200 μm) indicated that magnetic resonance targeted delivery of magnetic macrophages into the lungs had no adverse affects on the lung vasculature compared with delivery without targeting. Representative data are shown from one of two replicate experiments in which *n*=3 mice per group. s.e.m.’s are depicted and Student’s *t*-test *P*<0.75 compared magnetic resonance targeted with non-magnetic resonance targeted lungs.

MRT has particular application when tumours are difficult or impossible to remove surgically, as in the lung, brain, liver or spinal cord and may enable delivery of cell-based therapies to primary or metastatic tumours in such locations. In a second *in vivo* experiment, we used MRT to steer SPIO-labelled macrophages into the lungs of our mice bearing metastatic prostate tumours (Fig. 4). This was performed immediately after i.v. administration of 3 million macrophages. Mice not exposed to MRT, but exposed to the magnetic field of the scanner for the same length of time, were used as controls.

Flow cytometric analysis of enzymatically dispersed lungs showed the presence of significantly more human CD14+ macrophages following MRT than in the control group (17.7%±4 versus 4.4%±2.6, respectively) (Fig. 4a). This was also confirmed by histological and immunofluorescence staining of the lungs, where human CD68+ macrophages were detected in or close to the small metastatic deposits present within the lungs of mice following MRT (Fig. 4b; Supplementary Fig. 3a). These macrophages also stained positive for Prussian blue (iron) (Fig. 4b) and their iron content was also visible following haematoxylin and eosin staining (Supplementary Fig. 3b). We inspected the morphology of CD31+ blood vessels in the lungs following their uptake of SPIO-labelled macrophages with or without MRT (Fig. 4c). Due to the short T2/T2* of lung tissue, it was not possible to image the lung parenchyma with conventional ^1^H MRI techniques at high field for *in vivo* validation of increased uptake. Future technical developments may make this possible, for example, the use of hyperpolarized gases in the airspaces could be used as an indirect magnetic resonance signal detection method^28^. Nevertheless, in different organs or soft tissues, or on clinical systems, T2* imaging may have a place.

### MRT of oncolytic macrophages reduces tumour growth

**Figure 5:**
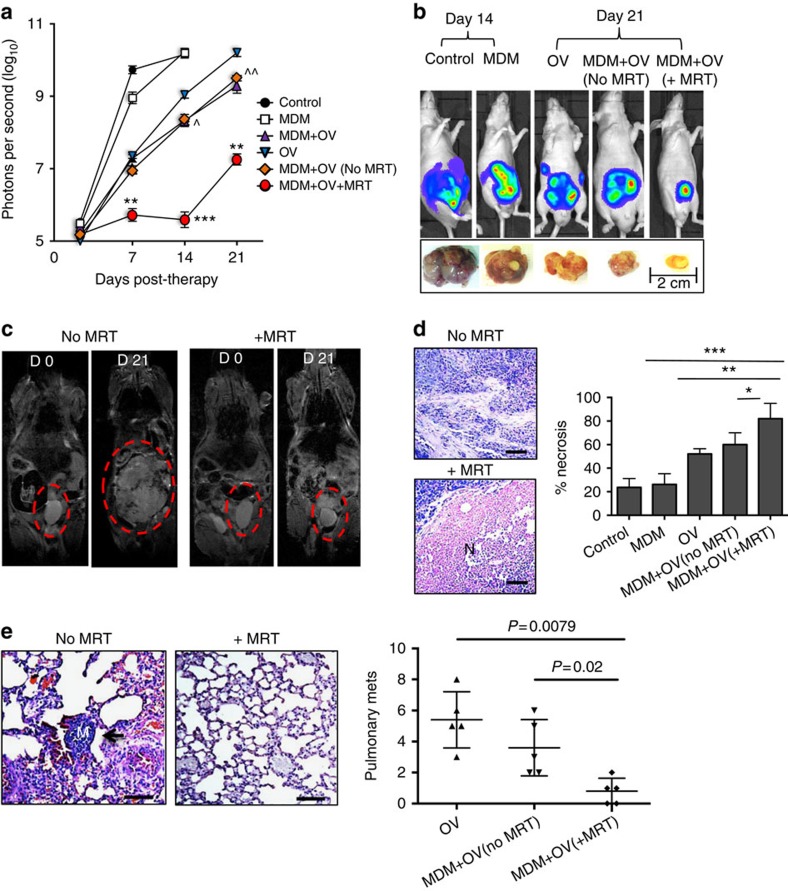
Magnetic targeting increases the anti-tumour effects of oncolytic macrophages. Tumour-bearing mice were administered with a single dose of human monocyte-derived macrophages (MDMs) carrying the oncolytic virus, HSV1716 (MDM+OV). These were divided into three groups of mice (each with five mice per group). One group underwent MRT to either the prostate gland or lungs (MDM+OV+MRT) for 1 h, another was exposed to the MRI scanner but with no MRT (MDM+OV no MRT) and the third (MDM+OV) did not enter the MRI scanner. Additional groups of mice received 100 μl of PBS (Control), a single dose of 1 × 10^7^ plaque-forming unit HSV1716 (OV) or 3 million untreated MDM. Mice were imaged weekly using the IVIS imaging system and, after 21 days, tumours and lungs were removed and processed for histology. (**a**) Tumour luminosity in *n*=5 mice per group showed MRT significantly improved the effect of OV-MDM on tumour growth. (**b**) Representative IVIS images and photographs of primary tumours following various treatments. (**c**) Representative RARE images for MDM+OV with or without MRT show marked difference in tumour size at the beginning and end of therapy. Representative images of haematoxylin- and eosin-stained sections from *n*=5 mice per group show (**d**) the area of necrosis (*N*) in primary tumours and (**e**) the number of metastases (see arrow, M, metastasis) in the lungs of mice receiving MDM+OV with or without MRT. Scale bar, 200 μm (**d**–**e**). Data shown are means±s.e.m. of *n*=5 mice per group. For the lung metastasis, quantitative analysis was carried out on 10 high-power fields (× 20 magnification) per tissue section. Comparisons between more than two groups were performed using one-way analysis of variance followed by *post hoc* Bonferroni test. *P<0.05; **P<0.001; ***P<0.0001 compared with MDM+OV+MRT to MDM+OV (no MRT) and ˆ*P*<0.05 and ˆˆ*P*<0.001 is comparing MDM+OV (no MRT) and free OV group.

In a final experiment to assess the therapeutic benefits of MRT, SPIO-loaded macrophages armed with the OV, Seprehvir (HSV1716), were administered to tumour-bearing mice. HSV1716 replication is supported by PC3 prostate cancer cells^29^ and here we show, for the first time, oncolysis in LNCaP cells in both hypoxic (0.5% O_2_) and normoxic (20% O_2_) conditions (Supplementary Fig. 4a). Seprehvir is readily taken up by macrophages and while uptake is significantly higher in normoxic culture conditions (Supplementary Fig. 4b), viral replication is greater in hypoxia and macrophage cell death is equally effective in a hypoxic environment (Supplementary Fig. 4c,d). In our *in vivo* model, tumour-bearing mice received either a single i.v. injection of OV-carrying macrophages (monocyte-derived macrophage (MDM)+OV), were placed in the static field of the scanner without MRT ‘MDM+OV(no MRT)’, or were exposed to the scanner with MRT (MDM+OV+MRT). For the purpose of comparison, ‘free’ OV was administered to a separate group of mice. Additional control groups of mice received either 100 μl saline treatment (control) or 3 million untreated macrophages (MDM) i.v. OV (1 × 10^7^ plaque-forming unit^29^ alone significantly delayed primary tumour growth for up to 7 days compared with mice receiving PBS or MDM only (Fig. 5a). This effect was significantly prolonged with macrophage-mediated delivery of Seprehvir (*P*<0.006 at day 14 and *P*<0.007 day 21, a one-way analysis of variance followed by *post hoc* Bonferroni test was used for statistical analysis) and concurred with our previous studies where macrophages carrying OV were more effective over viral infusion alone^17^,^18^. Of note, no differences were observed in mice receiving MDM+OV but were not exposed to the MRI scanner and MDM+OV (no MRT) where the latter is exposed to the scanner but with no steering. However, MRT of our macrophage virotherapy was not only better at reducing the growth of the primary tumours from day 7 onwards but also delayed primary tumour regrowth for the entire experiment (Fig. 5a). Bioluminescence of mice receiving macrophage OV therapy with or without MRT on the first day of treatment (day 0) and at the end of the experiment (day 21) showed this marked reduction of the primary tumour (Fig. 5a,b). This was confirmed visually on the MRI scans (Fig. 5c). Furthermore, tumours undergoing MRT following macrophage-delivered OV were significantly more necrotic than those not receiving MRT (Fig. 5d).

In the lungs, few metastases were detected in mice injected with PBS or MDM alone since mice had to be culled at day 14 (due to the large size of their primary tumours). Therefore, it was not valid to compare metastases in these control groups with the other experimental groups. However, as shown in Fig. 5e, the number of lung metastases was markedly reduced in mice that received magnetic resonance targeted, OV-bearing macrophages than in those which received the cells but not MRT.

## Discussion

**Figure 6:**
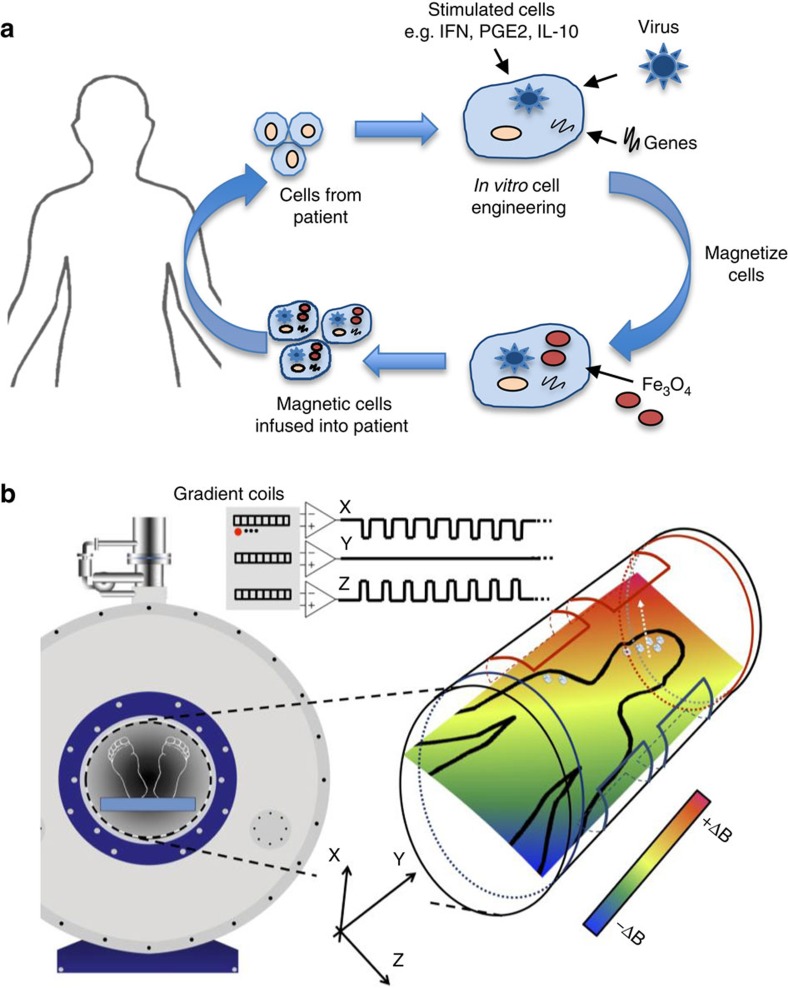
Principle of MRT to steer cell-based therapies to specific tissues. (**a**) The cells used for these studies are derived from monocytes isolated from patient blood. These cells are cultured in the presence of various stimuli to produce ‘therapeutic’ macrophages (for example, cytokines, therapeutic genes or viruses) and loaded with SPIOs before reinfusion back into the same patient. (**b**) The subject is then placed in the centre of an MRI scanner where linear spatial encoding magnetic gradients can be used to induce a force on a magnetized body. The magnetic cells injected into the bloodstream of the subject circulate and are targeted into the diseased organ/tissue under the influence of the applied magnetic field. Field map plots demonstrate that significant field gradients can be generated in various directions by the MRI gradient coils. The resulting magnetic field (d*B*/d*y* field) can steer magnetic cells towards the diseased tissue for increased cell uptake.

In this study, we show that an MRI scanner can be used to non-invasively steer cells to both primary and secondary tumours within the body leading to a significant improvement in therapeutic outcome. Moreover, relaxometry measurements suggest that standard MRI can then be used to monitor the efficacy of this therapy. While this study has focused on cell delivery to tumours, the technology could be used to target any cells (for example, mesenchymal stem cells and so on) to a given tissue in the body including non-phagocytic cell types, which could be ‘magnetized’ using SPIO-conjugated antibodies directed against proteins on their cell surface (Fig. 6).

The use of MRT, which exploits the magnetic field gradients within MRI systems to increase delivery of cells, is ideally suited to deep or superficial tissue^7^. The question of clinical translation is dependent on the ability to provide the same targeting force on a clinical MRI system. Clinical scanners, with high-performance magnetic field gradient systems of 300 mT m^−1^, are already in use and as such have the potential to produce similar forces^30^. Moreover, we were able to image the cell distributions following MRT, indicating the possibility for real-time image-guided targeting using an MRI system. These findings support the potential value of MRT with concomitant imaging for improved targeting of cells for therapy.

## Methods

### Isolation and culture of human macrophages

All patients donating blood gave informed consent to the Sheffield blood Transfusion Service and all procedures have been approved by the University of Sheffield Ethics Committee. Mononuclear cells were isolated from platelet-depleted buffy coats (Blood Transfusion Service, Sheffield, UK) using Ficoll-Paque Plus (Amersham Pharmacia, St Albans, UK).

In brief, 50 million monocytes were plated into T75 tissue culture flasks (NUNC, UK) and after 2 h non-adherent cells were removed. The remaining adherent cells were cultured over 7 days in IMDM (Lonza, UK) supplemented with 2 mmol l^−1^ L-glutamine, 100 U ml^−1^ penicillin, 100 μg ml^−1^ streptomycin and 2% human Ab serum (Lonza).

### Endothelial cell cultures

Human umbilical vein endothelial cells were obtained from Promocell, (Heidelberg, Germany) and used in the experiments up to passage 8. Cells (150,000) were seeded for 24 h onto collagen-coated (0.1 mg ml^−1^, human type IV) membranes containing a 5-μM pore polyethylene terephthalate membrane (Neuroprobe).

### Human multi-cellular tumour spheroids

Human prostate cancer cell line, LNCaP (ATCC CRL-1740), was seeded (5 × 10^3^) in 100 μl medium into each well of a 2% agarose (Sigma, Dorset, UK)-coated 96-well tissue culture plate. After 7–10 days, each well contained a tumour spheroid with an average diameter of 700–800 μm.

### Infection of primary macrophages

Day 3 MDMs were infected with a replication-deficient adenovirus (CMV-AdV5-GFP) at multiplicity of infection (MOI) 100. The E1A/B-deleted adenovirus, CMV-AdV5-GFP (driven by a CMV promoter), was isolated from a single plaque, expanded in 293 human embryonic kidney cells. All the viruses were purified by double caesium-gradient centrifugation, and titred by plaque assay on 293 cells with the titre expressed as plaque-forming units per cell. The MOI used in this study was previously optimized in macrophages and are described in ref. 17.

### Cellular uptake of magnetic nanoparticles by macrophages

MDMs (infected with Ad-CMV-GFP) were cultured overnight with 100 μg ml^−1^ SPIOs (25 nm) (Sigma-Aldrich, Poole, UK). SPIO accumulation in cells was previously assessed by flow cytometry and confirmed by attraction of the cells towards a magnet placed at the side of the culture dish as observed by light microscopy (Leica Microsystems UK Ltd). Cell viability following SPIO uptake by macrophages was also measured by flow cytometry and compared with cells that were not incubated with SPIOs using the DNA dye propidium iodide (Sigma). Comparisons made using an unpaired Student’s *t*-test revealed no statistically significant difference between the two groups *P*=0.4 (Fig. 1b) *N*=3.

### *In vitro* transendothelial flow assay

The TEM chamber was assembled as shown in Fig. 1a. SPIO-loaded MDM (1.5 × 10^5^ cells per ml in PBS+2% FCS) was flowed over the human umbilical vein endothelial cell monolayer at typical venous flow rates (1.1885, ml min^−1^) at a sheer stress of 1.4 dyn cm^−2^, this is equivalent to blood flow through post-capillary venules. The TEM chamber was positioned directly in the isocentre at ∼5-mm distal of a 7 T magnet (Bruker BioSpecAVANCEII, 310-mm bore, MRI system B/C 70/30). The flow in the chamber was in the –*z* direction (in and out of the magnet bore). We used pulsed gradients 2-ms on and 7-ms off as described by Reigler *et al.*^4^. To steer SPIOs into the chamber containing tumour spheroids, we applied a pulsed –*y* gradient at 50% strength to avoid over-heating (∼300 mT m^−1^) for 30 min. Post MRT, a ^1^H volume resonator (Bruker, 300 MHz, 1 kW max, outer diameter 118 mm per inner diameter 72 mm) allowed the capture of Fast low angle shot (FLASH) and RARE MRI images.

Spheroid infiltration by MDMs was then assessed using a fluorescent microscope to detect the GFP-positive cells and flow cytometry using enzymatically dispersed spheroids. To determine the iron content within SPIO-loaded macrophages, cell pellets were solubilized in 70% nitric acid for 7–14 days before analysis. Iron concentrations were quantified against a calibration standard iron solution (Fischer Scientific) by atomic emission spectroscopy using Varian Vista-M PX.

### Flow cytometric analysis

Single-cell suspensions were obtained by trypsinizing MDMs (including co-transduced MDMs). Cells were then incubated with for 30 min at 4 °C with mouse anti-CD14, 1:100 in PBS containing 1% bovine serum albumin (Sigma) to prevent nonspecific antibody binding. Alternatively, spheroids were digested using 0.25% trypsin/EDTA to separate the tumour cells and infiltrated macrophages and cell death was analysed by flow cytometry by adding propidium iodide (Sigma) to the cells immediately before running on the flow cytometer.

### Orthotopic prostate xenograft model

All mouse procedures were conducted in accordance with the UK Home Office Regulations under the Animals (Scientific Procedures) Act 1986 and the awarded project licence number under which these protocols were performed is PPL:40/3424. In addition, the University of Sheffield Animal Welfare and Ethical Review Body approved all the *in vivo* experiments used in this study. Male CD1 athymic mice (aged 7–8 weeks, stock number 000711) were used in these studies (Charles Rivers, UK). Animals were randomized before beginning the treatment schedule and were kept in ventilated cages with food and water provided *ad libitum*. Animal group sizes were calculated by power analysis. In general, a maximum of five animals per group were used unless otherwise stated. One million LNCaP:LUC:GFP cells (a gift from Professor Magnus Essand, Uppsala Sweden) were mixed 1:1 in Matrigel and injected into the dorsolateral prostate. Tumour size was determined by administering luciferin (Molecular Probes) followed by bioluminescent IVIS imaging and measuring the daily weights of the mice. Tumour uptake was monitored by bioluminescence imaging using the IVIS Lumina II imaging system (Caliper Life Sciences). This detects live luciferase-labelled tumour cells, enabling real-time monitoring of tumour growth and spread in the mice. The mice were injected intraperitoneally with 90 mg kg^−1^ D-luciferin (Caliper Life Sciences) dissolved in sterile water and anaesthetized using 2.5% isoflurane (Abbott Scandinavia AB) in 100% oxygen at 3.5 l min^−1^ (for induction) in the anaesthesia chamber of the imaging system. Mice were transferred to the dark box and isoflurane was lowered to 1.5%. Images were taken every 3 min as a sequence of 10 images for every group of mice, once a week. Automatic contour regions of interest were created, and the tumour sizes (or tumour radiance) were quantified as photons per second per square centimetre per steradian. Progression and spread of tumours were evaluated by calculating the tumour radiance values from inoculated mice in each group. Tumour-bearing mice were used in experiments ∼14 days following implantation or 21 days in the metastases model when the pulmonary tumours develop following orthotopic implantation of the tumour cells into the prostate^17^. Mice not developing tumours were excluded from the experiments (<5%). All mice were closely monitored and any displaying signs of rapid weight loss, excessively large tumours (>10^10^ photons per second, or 15 mm in diameter) or any pain/suffering/distress sufficient to impede natural behaviour were culled.

### Use of the MRI scanner to direct cell movement

Three million MDMs with or without SPIOs were administered via tail vein in 100 μl volume of PBS (*n*=5), control groups received 100 μl PBS (*n*=5) or 100 μl PBS containing 3 × 10^6^ macrophages without SPIOs (*n*=5). Immediately after MDM administration, mice were anaesthetized with gaseous isoflurane (Abbott, UK) and then secured within a magnet-compatible holding capsule and MRT was carried out immediately using a 7 T small bore magnet with a 660 mT m^−1^ gradient insert (Bruker BGA 12-S).

Mice were split into two groups of *n*=5. Group 1 was a time-matched control without MRT and group 2 underwent 1 h of MRT with gradients pulsed 2-ms on, 7-ms off at 50% total strength (300 mT m^−1^); and applied direction selected for coarse steering to the tumour site for the prostate (−*z* and −*y*) (Fig. 2a). For steering to the lungs (+*z* and −*y* gradients), the absence of an *x* gradient should ensure even distribution of magnetic particles in each lung.

The force on magnetically labelled cells is dependent on whether the SPIOs have become magnetically saturated. When unsaturated, the force is dependent on the magnetic susceptibility of the SPIOs, the magnetic field and also the magnetic field gradient^31^. However, once the SPIOs reach saturation, the force is no longer dependent on the magnetic susceptibility of the particle but the saturation magnetization and as such only the magnetic field gradient will affect the force applied to the cells^7^. SPIOs typically reach magnetic saturation well below 1 T, for example, in Riegler *et al.*^6^, where the SPIOs become saturated at around 300 mT, therefore, MRT is feasible on clinical MRI systems provided the same magnetic field gradient is used ∼300 mT m^−1^.

Following MRI steering, high-resolution RARE (retention time (TR)=4.2 s, TE=12 ms, RARE factor 8, 512 × 192, no averaging, 9 slices 1-mm thick) and gated FLASH (TR=8.9 ms, TE=1.2 ms, 24 reps, 128 × 128, flip angle (FA) 15°) images of the tumour (prostate only) were captured using a ^1^H volume resonator (Bruker, 300 MHz, 1 kW max, outer diameter 118 mm/inner diameter 72 mm). Once complete, relaxometry using multi-slice multi-echo (TE 10 ms, echo spacing 10 ms, 16 echoes, TR 2 s, matrix size 256 × 256) and multiple gradient echo (TE 2.5 ms, echo spacing 3.7 ms, 12 echoes, TR 10 s, matrix size 128 × 128, FA 90°) was performed to assess the transverse relaxation rates. After treatment, animals were killed and tissues, including tumours, kidney, liver, lungs and spleen, were either paraffin wax embedded and fixed for immunohistochemistry or analysed by flow cytometry to determine macrophage uptake (see Supplementary Files for details).

### Vascular permeability

A further study was performed to assess vascular permeability in mice. Mice were administered with 3 million SPIO-loaded MDM one group underwent MRT and the other remained in the scanner as described above. Immediately after targeting mice were injected i.v. with a 100 μl mixture of FITC-conjugated *Lycopersicon esculentum* (tomato) lectin (1 mg ml^−1^; Vector Laboratories) and *Ricinus communis* agglutinin I (2.5 mg ml^−1^; Vector Laboratories). Perfusion-fixation with 4% paraformaldehyde was performed 10 min following lectin administration. Harvested tissue was post-fixed in 4% paraformaldehyde, processed through graded sucrose and embedded in OCT medium (Tissue-Tek). Sections at ∼40 μm were counterstained with 4′,6-diamidino-2-phenylindole (0.05 mg ml^−1^, Invitrogen) and confocal image stacks were acquired by confocal microscopy (Nikon). Measurement of vascular volumes was performed on images from tumour-bearing mice with and without MRT targeting (*n*=3 mice per group and 5–10 fields of view).

In addition, vasculature leakage was also assessed using the contrast agent Gd-DPTA^31^,^32^. Mice receiving SPIO-loaded MDM with and without MRT were removed from the scanner and injected (via tail vein) with a 0.1 mmol Kg^−1^ dose of Gd-DPTA (Magnevist) (*N*=3 mice per group). Mice were then returned to the scanner and T1-weighted imaging (TR=100 ms, TE=3.7 ms, FA=30 degs, matrix size=256 × 256) was performed for 15 min (50 repetitions) post injection. Data were used to assess pooling of the contrast agent over time as an indicator of a leaky, damaged vasculature. Experiments were repeated with Gd-DPTA alone (no iron-labelled macrophages or MRT). Uptake of Gd-DTPA was monitored in tumour tissue over the 15-min period; any pooling would result in increased signal over this time period. We also monitored GD-DTPA uptake in the muscle surrounding the spinal cord (vertically away from the –*y* gradient targeted tumour region) as a control region where we expect no vascular disruption due to MRT. Direct comparisons in changes in signal intensity were made between these two regions to investigate any vascular damage in the targeted region. Finally, if there is no vascular damage, Gd-DTPA should enter the renal system; to confirm this in our groups, we imaged the kidneys.

### Therapeutic studies

HSV1716 *in vitro* studies are described in the accompanying Supplementary Files. *In vivo* studies were performed as follows. Tumour-bearing mice received tail-vein injections of either 3 million MDM alone or armed with Seprehvir at MOI 50, 1 × 10^7^ Seprehvir only or PBS (*n*=5 mice per group). Of note, three groups of mice were administered with MDM+OV, one group underwent MRT for 1 h, one sat in the MRI scanner but had no MRT (MDM+OV no MRT) and another group did not enter the MRI scanner (MDM+OV). Tumour size was monitored by IVIS Lumina II imaging (IVIS, Caliper Life Sciences). Animals were killed once tumours reached the maximum volume (>10^10^ photons per second) permitted by UK Home Office Regulations. Excised tissues including tumours, kidney, liver, lungs and spleen were embedded in OCT or paraffin wax for immunocytochemical/histological labelling studies.

### Tissue analysis

Tissues were divided into two; one part was formalin fixed for immunohistological analysis and the other was dissected free of adherent fibrous and fatty tissue and treated with collagenase. *Flow cytometry.* Cell viability was determined using LIVE/DEAD Fixable Violet Dead Cell Stain Kit (Invitrogen). All FACS data were analysed on an LSR II flow cytometer (BD Biosciences) using FlowJo software (Tree Star). *Histology*. Five-micron sections of all organs were incubated with specific antibodies for target antigens; for the vasculature we used CD31 (1:100; AbD Serotec) and for macrophages human CD68 (Dako, Ely, UK) at 1:100. A biotinylated secondary antibody system was used in conjunction with a streptavidin-conjugated horseradish peroxidase. Peroxidase activity was localized with diaminobenzidine (Vectastain Elite ABC kit, Vector Labs). To detect iron in the tumours (where cell densitites were high), sections were stained with Perls Prussian blue and counterstained with eosin for improved contrast. To detect cancer cells in the lungs, all lung sections were stained with epithelial cell adhesion molecule or haematoxylin and eosin. All immune-localization experiments were repeated on multiple tissue sections and included isotype-matched controls for determination of background staining. To assess necrosis, the area of necrosis within the whole-tumour section was determined visually, and the proportion of necrotic nonviable tumour areas over the whole section was calculated using ImageJ software (National Institute of Health). For each group, the mean percentage of necrosis and standard error were calculated. The results are presented as the mean tumour necrosis (%) for all tumours (five slices per each tumour) in each treatment group. *Immunofluorescence*. harvested tissue was post-fixed in 4% paraformaldehyde, processed through graded sucrose and embedded in OCT medium (Tissue-Tek) and stored at −80 °C. Frozen sections were dried for 10 min at room temperature and blocked in 5% horse serum+0.5% saponin in PBS for 30 min. Sections at ∼40 μm were stained with anti-GFP 1:200 (ab290 abcam, UK) and CD68 (1:100) for 1 h and then secondary antibodies donkey anti-rabbit alexa fluor 488 and goat anti-mouse 540 at 1:100 dilution (Invitrogen, Paisley, UK) and finally counterstained with prolong gold-antifade mountant with 4′,6-diamidino-2-phenylindole (0.05 mg ml^−1^, Invitrogen). Images of macrophage infiltration into primary and pulmonary LNCaP tumours were captured using a spinning disc confocal microscope (Olympus IX81, PerkinElmer, UK). Confocal image Z-stacks of tumours were captured at 1-μm increments at × 20 magnification.

### Statistical analysis

Data are means±s.e.m. (Prism 5; GraphPad Software). Two-tailed Student’s *t*-test was used to analyse the statistical significance of the data unless otherwise stated. Differences were termed significant with a *P* value of <0.05.

## Additional information

**How to cite this article:** Muthana, M. *et al.* Directing cell therapy to anatomic target sites *in vivo* with magnetic resonance targeting. *Nat. Commun.* 6:8009 doi: 10.1038/ncomms9009 (2015).

## Supplementary Material

Supplementary Information Supplementary Figures 1-4
