# Supplementary material for: Directing cell therapy to anatomic target sites in vivo with magnetic resonance targeting
Source: Nat Commun. 2015 Aug 18;6:8009. doi: 10.1038/ncomms9009 (PMC4568295; doi:10.1038/ncomms9009)
Supplement: Supplementary Information — Supplementary Figures 1-4 [file ncomms9009-s1.pdf]

Supplementary Figure 1

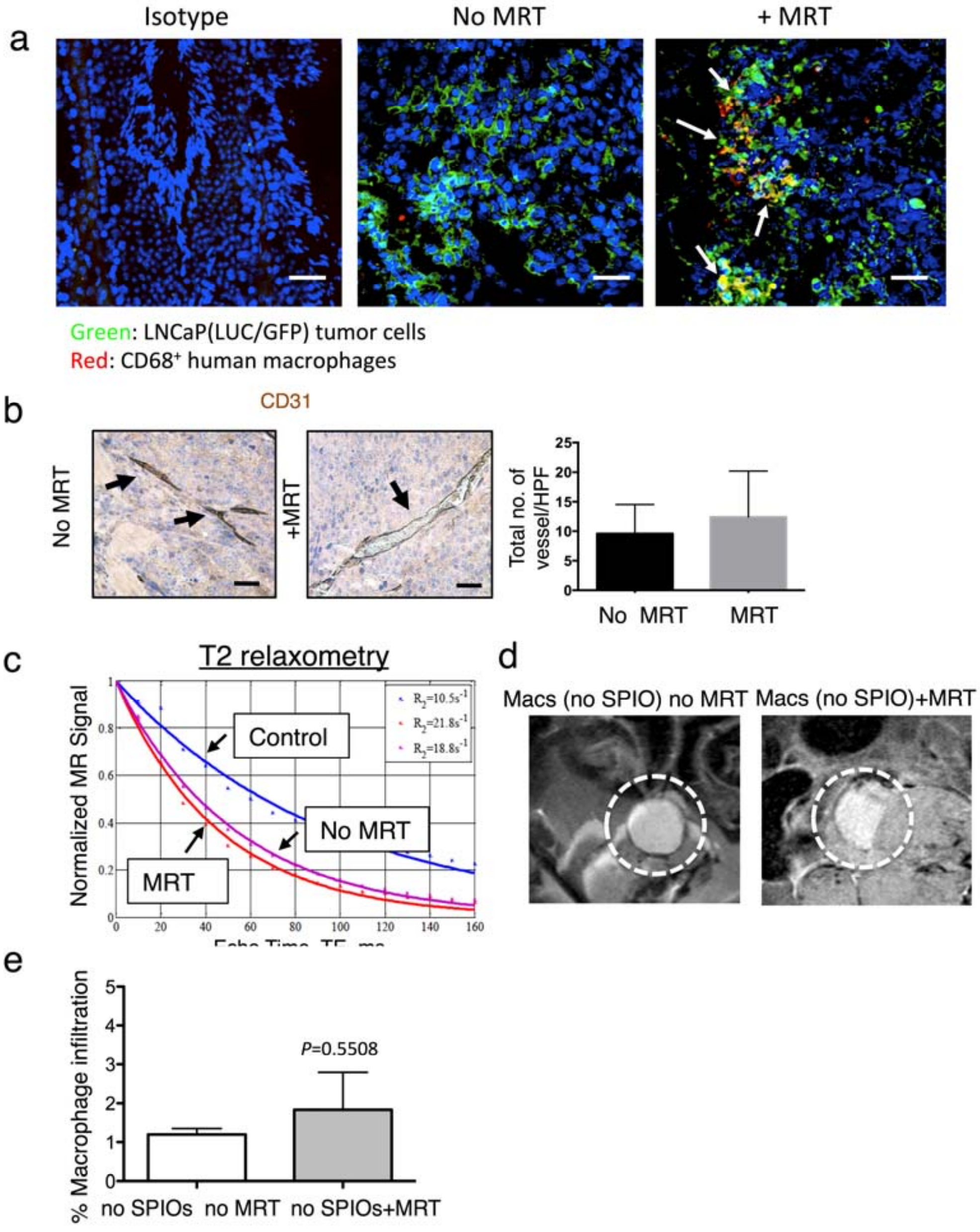

Supplementary Figure 1. Magnetic macrophages were steered into primary prostate tumors using magnetic fields generated in an MRI scanner. Three million magnetically-labelled macrophages were administered via i.v. injection and mice were then placed into the isocentre of a 7T MRI scanner. Subjects were split into 2 groups. Group 1 were imaged after 1 hour (no MRT). Group 2 underwent MRT. Representative immunofluorescence images from n=5 mice/group show tumor sections stained with anti-GFP (green) and CD68 (red), this revealed the presence of increased infiltrating macrophages in mice that received MRT compared to mice receiving no MRT (see arrows). Representative images of CD31 labelled

vessels (see arrows) from n=5 mice/group and the number of vessels per high power field per view was recorded (b). Steering of macrophages into the tissue using MRT had no significant effect on vessel numbers in tumors (p=0.5165) compared to mice who received no MRT. Representative MRI images for each group following MRT into tumors show a qualitative decrease in signal and this was confirmed by analysis of the transverse relaxation rate in both groups (c). Group 2 shows an increased decay rate over group 1. From this we can estimate the best echo time for looking at signal differences with MRI is around 60ms – MRI steering leads to a 10% decrease in signal at this echo time. This significant signal decrease suggests the presence of increased levels of iron in-group 2. The normal decay rate of tumor tissue is also shown for comparison (control). This experiment was repeated but using macrophages without SPIOs (N=3 mice per group). Very little distortion was visible in the MRI images of tumors in (d), indicating low uptake of non-magnetic macrophages and this was confirmed by FACS analysis of collagenase treated tissue (e). Data are presented are the means ± SEM. All data shown are the means ± SEM of n=5/group and two-tailed student's t-test were applied for statistical analysis. Bars in panel a = 70μm and in panel b= 200μm.

Supplementary Figure 2

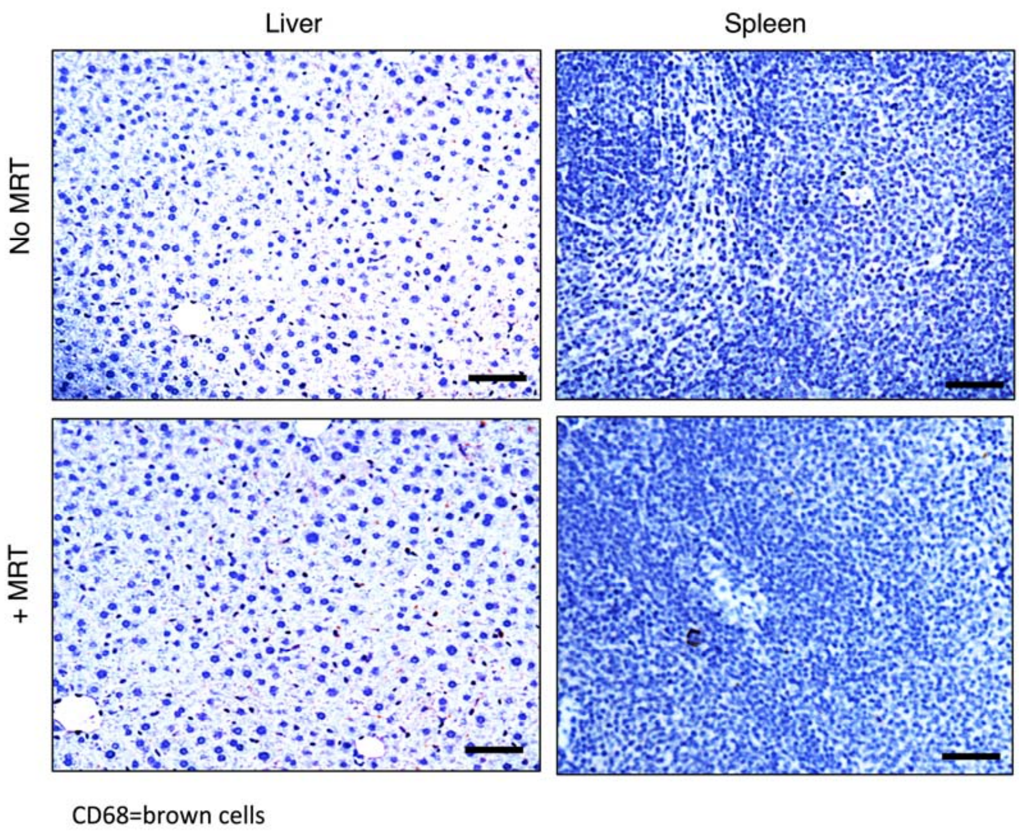

Supplementary Figure 2. Magnetic macrophages were detected in very low numbers in other tissues/organs. MRI steering of magnetic macrophages into tumors resulted in very few macrophages localising to other tissues. This was determined by histological staining of paraffin-wax embedded sections of tissues and organs removed post-mortem. Here we show representative images from n=5 mice/group of the liver and spleen taken from tumor-bearing mice injected with SPIO-loaded macrophages and then subjected to MRT or no MRT. In both these tissues we detected virtually no human macrophages (<2% in whole tissue section) following staining with anti-CD68. Bars = 100µm.

Supplementary Figure 3

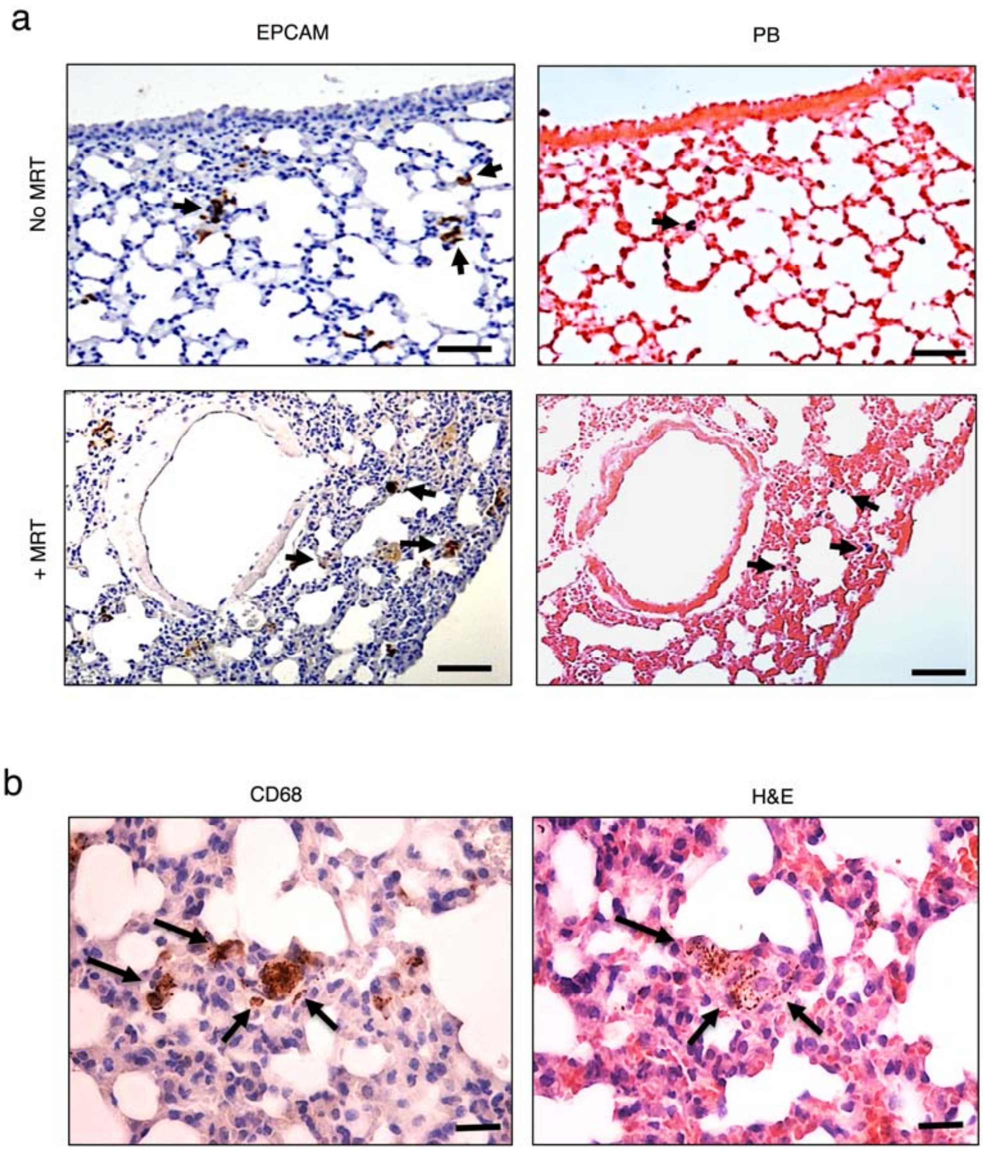

Supplementary Figure 3. Magnetic macrophages were steered into areas of pulmonary metastasis using MRT. This was confirmed by immunohistochemical staining of wax-embedded sequential sections of lung tissue with an antibody to human EPCAM (brown – see arrows) which detects human prostate tumor cells, and Prussian Blue (PB) to detect the iron-positive human macrophages (see arrows). Representative images from n=3 mice/group show that macrophages positive for PB were detected in close proximity to the metastatic deposits (see arrows) within the lungs of mice following MRI steering (a). Representative lung

sections from n=3 mice/group show CD68 human macrophages (see arrows) were detected in the lungs of mice (b). Of note, the iron with macrophages targeted to the lungs by MRT (arrows) was also visible following H&E staining (b). Bars in panel a = 200μm and Bars in b= 50μm.

Supplementary Figure 4

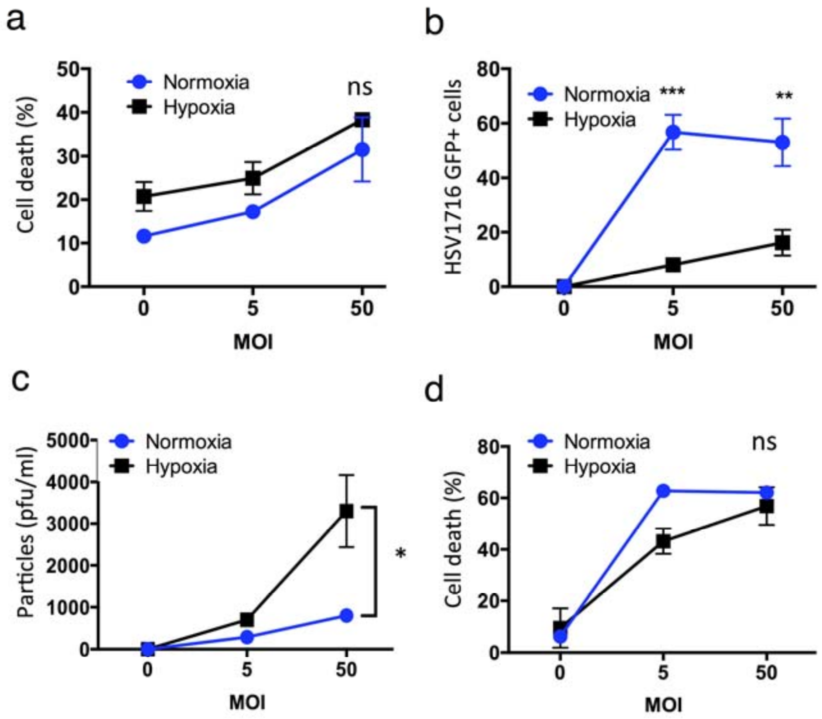

Supplementary Figure 4. HSV1716 induces LNCaP and macrophage oncolysis. HSV1716:GFP was added to cultures of LNCaP cells incubated in normoxic (20% O_2) and hypoxic (0.5% O_2) culture conditions. Tumour cell death was assessed by flow cytometry using propidium iodide and increased over uninfected cells (a). This was dose dependent and in normoxia at MOI 5 $p< 0.03$ at MOI50 $p<0.001$ & in hypoxia at MOI5 $p<0.01$ at MOI50 $p<0.001$. No statistical significance was observed between normoxic and hypoxic conditions at both MOI 5 and 50. HSV1716 is effectively taken up by MDM at MOI 5 and 50 as assessed by flow cytometry 48h post infection. Normoxic culture conditions resulted in significantly more GFP expressing macrophages at MOI 5 (p< 0.0004) and MOI 50 ($p <$ 0.001) compared to hypoxic conditions (b) but interestingly the concentration of HSV1716 (PFU/ml) detected in macrophage supernatants 96 h following infection was greater at both MOI5 and 50 in hypoxia compared to normoxic conditions (c). Finally, macrophage cell death was equally induced in both normoxia and hypoxia ($p<0.2$) following infection with HSV1716 (d). Data are the mean ± SEM of 5 independent experiments. For statistical analysis one-way analysis of variance (ANOVA) Bonferroni tests were applied throughout.
